# (2-{[2-(1*H*-Benzimidazol-2-yl-κ*N* ^3^)phen­yl]imino­methyl-κ*N*}-5-methyl­phenolato-κ*O*)chloridozinc(II)

**DOI:** 10.1107/S1600536811030170

**Published:** 2011-08-02

**Authors:** Naser Eltaher Eltayeb, Siang Guan Teoh, Suchada Chantrapromma, Hoong-Kun Fun

**Affiliations:** a School of Chemical Sciences, Universiti Sains Malaysia, 11800 USM, Penang, Malaysia; b Department of Chemistry, Faculty of Pure and Applied Sciences, International University of Africa, Sudan; c Crystal Materials Research Unit, Department of Chemistry, Faculty of Science, Prince of Songkla University, Hat-Yai, Songkhla 90112, Thailand; d X-ray Crystallography Unit, School of Physics, Universiti Sains Malaysia, 11800 USM, Penang, Malaysia

## Abstract

In the title mononuclear complex, [Zn(C_21_H_16_N_3_O)Cl], the Zn^II^ ion is coordinated in a distorted tetra­hedral geometry by two benzimidazole N atoms and one phenolate O atom from the tridentate Schiff base ligand and a chloride ligand. The benzimidazole ring system forms dihedral angles of 26.68 (9) and 56.16 (9)° with the adjacent benzene ring and the methyl­phenolate group benzene ring, respectively. In the crystal, mol­ecules are linked by N—H⋯Cl hydrogen bonds into chains along [100]. Furthermore, weak C—H⋯O and C—H⋯π inter­actions, in addition to π–π inter­actions with centroid–centroid distances in the range 3.5826 (13)–3.9681 (13) Å, are also observed.

## Related literature

For standard bond-length data, see: Allen *et al.* (1987 ▶). For background to benzimidazoles and their applications, see: Chassaing *et al.* (2008 ▶); Kucukbay *et al.* (2003 ▶); Podunavac-Kuzmanovic & Cvetkovic (2010 ▶); Podunavac-Kuzmanovic *et al.* (1999 ▶); Podunavac-Kuzmanovic & Markov (2006 ▶); Xue *et al.* (2011 ▶). For related structures, see: Eltayeb *et al.* (2007 ▶, 2009 ▶); Eltayeb, Teoh, Chantrapromma & Fun (2011 ▶); Eltayeb, Teoh, Yeap & Fun (2011 ▶); Maldonado-Rogado *et al.* (2007 ▶); Tong & Ye (2004 ▶). For the stability of the temperature controller used in the data collection, see: Cosier & Glazer, (1986 ▶).
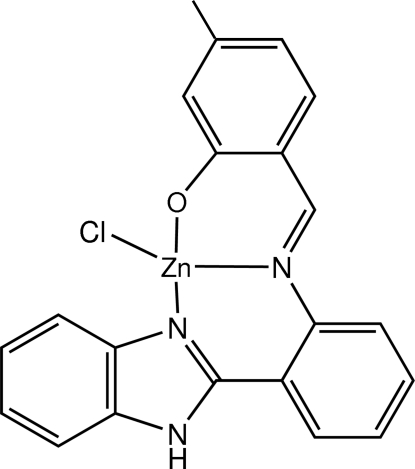


## Experimental

### 

#### Crystal data

[Zn(C_21_H_16_N_3_O)Cl]
*M* *_r_* = 427.21
Monoclinic, 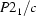

*a* = 8.6338 (1) Å
*b* = 19.4952 (2) Å
*c* = 10.9687 (1) Å
β = 99.675 (1)°
*V* = 1819.97 (3) Å^3^
*Z* = 4
Mo *K*α radiation
μ = 1.51 mm^−1^
*T* = 100 K
0.26 × 0.18 × 0.09 mm

#### Data collection

Bruker APEXII CCD area-detector diffractometer
Absorption correction: multi-scan (*SADABS*; Bruker, 2005 ▶) *T*_min_ = 0.694, *T*_max_ = 0.878
22729 measured reflections
5678 independent reflections
3773 reflections with *I* > 2σ(*I*)
*R*_int_ = 0.042

#### Refinement

*R*[*F* ^2^ > 2σ(*F* ^2^)] = 0.042
*wR*(*F* ^2^) = 0.088
*S* = 1.03
5678 reflections
249 parameters
H atoms treated by a mixture of independent and constrained refinement
Δρ_max_ = 0.64 e Å^−3^
Δρ_min_ = −0.39 e Å^−3^

### 

Data collection: *APEX2* (Bruker, 2005 ▶); cell refinement: *SAINT* (Bruker, 2005 ▶); data reduction: *SAINT*; program(s) used to solve structure: *SHELXTL* (Sheldrick, 2008 ▶); program(s) used to refine structure: *SHELXTL*; molecular graphics: *SHELXTL*; software used to prepare material for publication: *SHELXTL* and *PLATON* (Spek, 2009 ▶).

## Supplementary Material

Crystal structure: contains datablock(s) global, I. DOI: 10.1107/S1600536811030170/lh6598sup1.cif

Structure factors: contains datablock(s) I. DOI: 10.1107/S1600536811030170/lh6598Isup2.hkl

Additional supplementary materials:  crystallographic information; 3D view; checkCIF report

## Figures and Tables

**Table 1:** Hydrogen-bond geometry (Å, °) *Cg*1 and *Cg*2 are the centroids of the C15–C20 and C8–C13 rings, respectively.

*D*—H⋯*A*	*D*—H	H⋯*A*	*D*⋯*A*	*D*—H⋯*A*
N2—H1N1⋯Cl1^i^	0.75 (3)	2.53 (3)	3.2352 (19)	157 (2)
C2—H2*A*⋯O1^ii^	0.93	2.59	3.425 (3)	149
C12—H12*A*⋯*Cg*1^iii^	0.93	2.96	3.762 (3)	145
C21—H21*C*⋯*Cg*2^iv^	0.96	2.92	3.741 (3)	144

Symmetry codes: (i) 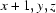; (ii) 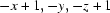; (iii) 
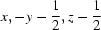
; (iv) 
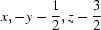
.

**Fig. 1.**
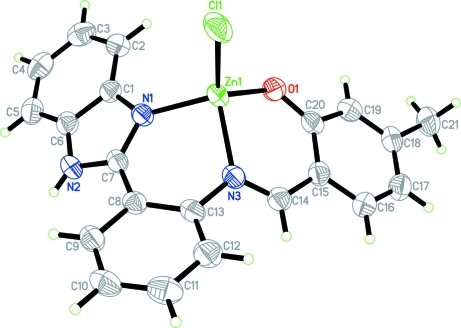
The molecular structure of the title compound, with 50% probability displacement ellipsoids.

**Fig. 2.**
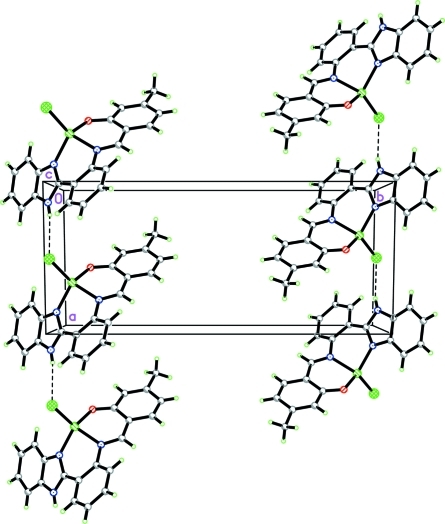
The crystal packing of the title compound viewed approximately along the c axis. N—H···Cl hydrogen bonds are shown as dashed lines.

## supplementary crystallographic information

### Comment

Benzimidazole compounds and their complexes have been found to show diverse biological activity (Chassaing *et al.*, 2008; Kucukbay *et al.*, 2003; Podunavac-Kuzmanovic & Cvetkovic, 2010; Podunavac-Kuzmanovic *et al.*, 1999; Podunavac-Kuzmanovic & Markov, 2006) including inhibition against enteroviruses (Xue *et al.*, 2011). Our ongoing structural studies involves benzimidazoles (Eltayeb *et al.*, 2007, 2009; Eltayeb, Teoh, Yeap & Fun, 2011) and their complexes (Eltayeb, Teoh, Chantrapromma & Fun, 2011). In the preparation of the title complex (I), 2-(2-aminophenyl)-1*H*-benzimidazole undergoes a condensation reaction with 2-hydroxy-4-methylbenzaldehyde to give a Schiff base ligand and forming the zinc(II) complex.

Complex (I) is a mononuclear zinc(II) complex (Fig. 1) in which the environment around the Zn^II^ ion is a distorted tetrahedral geometry and the Zn^II^ ion is four-coordinated by the two benzimidazole N atoms, one phenolate O atom and a Cl ligand. In the complex, the Schiff base ligand acts as a tridentate ligand. The bond angles around the central metal zinc(II) show large deviations from ideal tetrahedral geometry [O1-Zn1-Cl1 = 115.14 (5)°, N1-Zn1-Cl1 = 111.84 (5)°, N3-Zn1-Cl1 = 120.39 (6)°; and the bite angles N1–Zn1-N3 = 90.39 (7)° and O1-Zn1-N3 = 95.00 (7)°]. The Zn-N [1.9954 (17) and 2.2092 (18) Å], Zn-O [1.9137 (15) Å] and Zn-Cl [2.2249 (7) Å] bond lengths are comparable to those of similar Zn(II) benzimidazole complexes (Eltayeb, Teoh, Chantrapromma & Fun, 2011; Maldonado-Rogado *et al.*, 2007; Tong & Ye, 2004). The benzimidazole ring system (C1–C7/N1–N2) is planar with an *r.m.s*. deviation of 0.0074 (2) Å and the largest deviation of 0.029 (2) Å for atom N1. The benzimidazole ring system forms dihedral angles of 26.68 (9) and 56.16 (9)° with the C8–C13 and C15–C20 rings, respectively. The dihedral angle between the C8–C13 and C15–C20 benzene rings is 35.26 (11)°. The bond lengths of ligand are within normal ranges (Allen *et al.*, 1987).

In the crystal structure of (I) as shown Fig. 2, the molecules are linked through N—H···Cl hydrogen bonds (Table 1) into chains along the *a* axis. C—H···O and C—H···π weak interactions (Table 1) are also present. π–π interactions were also observed with centroid···centroid distances: *Cg*1···*Cg*2^v^ = 3.6134 (13) Å; *Cg*1···*Cg*3^vi^ = 3.9681 (13) Å and *Cg*2···*Cg*2^v^ = 3.5826 (13) Å; *Cg*1, *Cg*2 and *Cg*3 are the centroids of the C1/C6–C7/N1–N2, C1–C6 and C8–C13 rings, respectively [symmetry codes: (v) 2-x, -x, 1-z; (vi) 2-x, -y, 2-z].

### Experimental

The title compound was synthesized by adding 2-hydroxy-4-methylbenzaldehyde (0.136 g, 1.0 mmol) to a solution of 2-(2-aminophenyl)-1*H*-benzimidazole (0.209 g, 1.0 mmol) in ethanol (30 mL). The color of the resulting solution was pale-yellow. Upon adding zinc chloride (0.136 g, 1.0 mmol), the color of the solution turned golden-yellow. The mixture was refluxed with stirring for 3 hrs. Yellow block-shaped single crystals of the title compound suitable for *x*-ray structure determination were obtained from ethanol by slow evaporation at room temperature after several days.

### Refinement

H atom attached to N2 was located in a difference map and refined isotropically. The remaining H atoms were positioned geometrically and allowed to ride on their parent atoms, with d(C-H) = 0.93 Å for aromatic and CH; and 0.96 Å for CH_3_. The *U*_iso_ values were constrained to be 1.5*U*_eq_ of the carrier atom for methyl H atoms and 1.2*U*_eq_ for the remaining H atoms. A rotating group model was used for the methyl groups. The highest residual electron density peak is located at 0.89 Å from Zn1 and the deepest hole is located at 0.74 Å from Zn1.

### Crystal data

**Table:**

[Zn(C_21_H_16_N_3_O)Cl]	*F*(000) = 872
*M**_r_* = 427.21	*D*_x_ = 1.559 Mg m^−^^3^
Monoclinic, *P*2_1_/*c*	Mo *K*α radiation, λ = 0.71073 Å
Hall symbol: -P 2ybc	Cell parameters from 5678 reflections
*a* = 8.6338 (1) Å	θ = 2.1–30.7°
*b* = 19.4952 (2) Å	µ = 1.51 mm^−^^1^
*c* = 10.9687 (1) Å	*T* = 100 K
β = 99.675 (1)°	Block, yellow
*V* = 1819.97 (3) Å^3^	0.26 × 0.18 × 0.09 mm
*Z* = 4	

### Data collection

**Table:**

Bruker APEXII CCD area-detector diffractometer	5678 independent reflections
Radiation source: sealed tube	3773 reflections with *I* > 2σ(*I*)
graphite	*R*_int_ = 0.042
φ and ω scans	θ_max_ = 30.7°, θ_min_ = 2.1°
Absorption correction: multi-scan (*SADABS*; Bruker, 2005)	*h* = −12→12
*T*_min_ = 0.694, *T*_max_ = 0.878	*k* = −28→26
22729 measured reflections	*l* = −12→15

### Refinement

**Table:**

Refinement on *F*^2^	Primary atom site location: structure-invariant direct methods
Least-squares matrix: full	Secondary atom site location: difference Fourier map
*R*[*F*^2^ > 2σ(*F*^2^)] = 0.042	Hydrogen site location: inferred from neighbouring sites
*wR*(*F*^2^) = 0.088	H atoms treated by a mixture of independent and constrained refinement
*S* = 1.03	*w* = 1/[σ^2^(*F*_o_^2^) + (0.0283*P*)^2^ + 0.6885*P*] where *P* = (*F*_o_^2^ + 2*F*_c_^2^)/3
5678 reflections	(Δ/σ)_max_ = 0.001
249 parameters	Δρ_max_ = 0.64 e Å^−^^3^
0 restraints	Δρ_min_ = −0.39 e Å^−^^3^

### Special details

**Table:**

Experimental. The crystal was placed in the cold stream of an Oxford Cryosystems Cobra open-flow nitrogen cryostat (Cosier & Glazer, 1986) operating at 120.0 (1) K.
Geometry. All esds (except the esd in the dihedral angle between two l.s. planes) are estimated using the full covariance matrix. The cell esds are taken into account individually in the estimation of esds in distances, angles and torsion angles; correlations between esds in cell parameters are only used when they are defined by crystal symmetry. An approximate (isotropic) treatment of cell esds is used for estimating esds involving l.s. planes.
Refinement. Refinement of F^2^ against ALL reflections. The weighted R-factor wR and goodness of fit S are based on F^2^, conventional R-factors R are based on F, with F set to zero for negative F^2^. The threshold expression of F^2^ > 2sigma(F^2^) is used only for calculating R-factors(gt) etc. and is not relevant to the choice of reflections for refinement. R-factors based on F^2^ are statistically about twice as large as those based on F, and R- factors based on ALL data will be even larger.

### Fractional atomic coordinates and isotropic or equivalent isotropic displacement parameters (Å^2^)

**Table:**

	*x*	*y*	*z*	*U*_iso_*/*U*_eq_	
Zn1	0.66403 (3)	0.062335 (13)	0.71581 (2)	0.03608 (9)	
Cl1	0.50460 (7)	0.00213 (4)	0.81468 (7)	0.05647 (19)	
O1	0.56252 (18)	0.11090 (8)	0.57283 (14)	0.0414 (4)	
N1	0.86821 (19)	0.01439 (9)	0.71573 (16)	0.0330 (4)	
N2	1.1246 (2)	−0.00194 (10)	0.76330 (18)	0.0375 (4)	
N3	0.7792 (2)	0.14557 (9)	0.79697 (16)	0.0349 (4)	
C1	0.9123 (2)	−0.03570 (11)	0.63665 (19)	0.0336 (5)	
C2	0.8211 (3)	−0.07198 (12)	0.5421 (2)	0.0413 (5)	
H2A	0.7133	−0.0651	0.5221	0.050*	
C3	0.8977 (3)	−0.11859 (13)	0.4794 (2)	0.0504 (6)	
H3A	0.8401	−0.1447	0.4167	0.060*	
C4	1.0616 (3)	−0.12755 (13)	0.5080 (2)	0.0496 (6)	
H4A	1.1098	−0.1590	0.4628	0.060*	
C5	1.1522 (3)	−0.09105 (12)	0.6007 (2)	0.0445 (6)	
H5A	1.2606	−0.0967	0.6190	0.053*	
C6	1.0740 (3)	−0.04558 (11)	0.6655 (2)	0.0358 (5)	
C7	0.9989 (2)	0.03287 (11)	0.79017 (19)	0.0318 (5)	
C8	1.0111 (2)	0.08132 (11)	0.89379 (19)	0.0332 (5)	
C9	1.1357 (3)	0.07388 (12)	0.9935 (2)	0.0388 (5)	
H9A	1.2078	0.0386	0.9919	0.047*	
C10	1.1532 (3)	0.11775 (13)	1.0936 (2)	0.0456 (6)	
H10A	1.2370	0.1123	1.1582	0.055*	
C11	1.0461 (3)	0.16957 (14)	1.0971 (2)	0.0520 (7)	
H11A	1.0567	0.1986	1.1653	0.062*	
C12	0.9230 (3)	0.17893 (13)	1.0006 (2)	0.0451 (6)	
H12A	0.8517	0.2144	1.0042	0.054*	
C13	0.9045 (2)	0.13597 (11)	0.89831 (19)	0.0351 (5)	
C14	0.7407 (2)	0.20702 (12)	0.7581 (2)	0.0374 (5)	
H14A	0.7912	0.2430	0.8042	0.045*	
C15	0.6287 (2)	0.22529 (11)	0.6517 (2)	0.0362 (5)	
C16	0.6017 (3)	0.29611 (12)	0.6307 (2)	0.0438 (6)	
H16A	0.6503	0.3273	0.6890	0.053*	
C17	0.5065 (3)	0.32035 (13)	0.5274 (2)	0.0470 (6)	
H17A	0.4893	0.3673	0.5172	0.056*	
C18	0.4349 (3)	0.27452 (12)	0.4370 (2)	0.0411 (5)	
C19	0.4615 (3)	0.20532 (12)	0.4549 (2)	0.0408 (5)	
H19A	0.4170	0.1753	0.3928	0.049*	
C20	0.5529 (2)	0.17729 (12)	0.5628 (2)	0.0365 (5)	
C21	0.3284 (3)	0.29989 (14)	0.3235 (2)	0.0541 (7)	
H21A	0.3299	0.2681	0.2569	0.081*	
H21B	0.3640	0.3440	0.3006	0.081*	
H21C	0.2232	0.3038	0.3405	0.081*	
H1N1	1.208 (3)	0.0047 (13)	0.793 (2)	0.051 (8)*	

### Atomic displacement parameters (Å^2^)

**Table:**

	*U*^11^	*U*^22^	*U*^33^	*U*^12^	*U*^13^	*U*^23^
Zn1	0.02462 (12)	0.03720 (15)	0.04474 (16)	0.00021 (11)	0.00096 (10)	0.00267 (12)
Cl1	0.0295 (3)	0.0641 (4)	0.0770 (5)	−0.0005 (3)	0.0123 (3)	0.0210 (4)
O1	0.0393 (8)	0.0357 (9)	0.0447 (9)	−0.0003 (7)	−0.0063 (7)	0.0032 (7)
N1	0.0269 (8)	0.0347 (10)	0.0367 (9)	−0.0008 (7)	0.0029 (7)	0.0039 (8)
N2	0.0252 (9)	0.0420 (11)	0.0433 (11)	0.0027 (9)	0.0000 (8)	0.0006 (9)
N3	0.0280 (8)	0.0382 (10)	0.0372 (10)	0.0010 (8)	0.0018 (7)	−0.0020 (8)
C1	0.0344 (11)	0.0308 (11)	0.0357 (11)	0.0002 (9)	0.0062 (9)	0.0037 (9)
C2	0.0374 (12)	0.0410 (14)	0.0442 (13)	−0.0029 (10)	0.0030 (10)	0.0003 (11)
C3	0.0646 (17)	0.0414 (14)	0.0440 (14)	−0.0089 (13)	0.0054 (12)	−0.0046 (12)
C4	0.0651 (17)	0.0369 (14)	0.0507 (15)	0.0040 (12)	0.0211 (13)	−0.0006 (11)
C5	0.0437 (13)	0.0407 (13)	0.0505 (14)	0.0077 (11)	0.0118 (11)	0.0068 (12)
C6	0.0337 (11)	0.0356 (12)	0.0388 (12)	0.0015 (9)	0.0079 (9)	0.0054 (10)
C7	0.0262 (9)	0.0350 (11)	0.0340 (11)	0.0007 (9)	0.0043 (8)	0.0070 (9)
C8	0.0288 (10)	0.0352 (12)	0.0356 (11)	−0.0040 (9)	0.0055 (8)	0.0050 (9)
C9	0.0322 (11)	0.0425 (13)	0.0401 (12)	−0.0009 (10)	0.0014 (9)	0.0066 (10)
C10	0.0396 (12)	0.0573 (16)	0.0365 (12)	−0.0095 (12)	−0.0038 (10)	0.0043 (11)
C11	0.0538 (15)	0.0623 (17)	0.0377 (13)	−0.0100 (14)	0.0017 (11)	−0.0110 (12)
C12	0.0430 (13)	0.0491 (15)	0.0429 (13)	0.0009 (11)	0.0061 (10)	−0.0089 (11)
C13	0.0281 (10)	0.0392 (12)	0.0367 (11)	−0.0046 (9)	0.0021 (8)	0.0006 (10)
C14	0.0331 (11)	0.0389 (13)	0.0400 (12)	0.0001 (10)	0.0060 (9)	−0.0046 (10)
C15	0.0327 (11)	0.0366 (12)	0.0398 (12)	0.0038 (9)	0.0075 (9)	0.0011 (10)
C16	0.0417 (13)	0.0394 (13)	0.0497 (14)	0.0029 (11)	0.0056 (11)	−0.0042 (11)
C17	0.0442 (13)	0.0387 (14)	0.0581 (15)	0.0063 (11)	0.0084 (12)	0.0055 (12)
C18	0.0344 (11)	0.0458 (14)	0.0440 (13)	0.0070 (10)	0.0089 (10)	0.0078 (11)
C19	0.0370 (12)	0.0460 (14)	0.0385 (12)	−0.0024 (10)	0.0036 (9)	−0.0001 (10)
C20	0.0272 (10)	0.0426 (13)	0.0402 (12)	0.0003 (9)	0.0070 (9)	−0.0010 (10)
C21	0.0474 (14)	0.0585 (17)	0.0544 (15)	0.0114 (13)	0.0022 (12)	0.0111 (13)

### Geometric parameters (Å, °)

**Table:**

Zn1—O1	1.9137 (15)	C8—C13	1.415 (3)
Zn1—N1	1.9954 (17)	C9—C10	1.380 (3)
Zn1—N3	2.0292 (18)	C9—H9A	0.9300
Zn1—Cl1	2.2249 (7)	C10—C11	1.375 (3)
O1—C20	1.300 (3)	C10—H10A	0.9300
N1—C7	1.327 (2)	C11—C12	1.380 (3)
N1—C1	1.401 (3)	C11—H11A	0.9300
N2—C7	1.354 (3)	C12—C13	1.388 (3)
N2—C6	1.381 (3)	C12—H12A	0.9300
N2—H1N1	0.75 (3)	C14—C15	1.430 (3)
N3—C14	1.296 (3)	C14—H14A	0.9300
N3—C13	1.427 (2)	C15—C16	1.413 (3)
C1—C2	1.386 (3)	C15—C20	1.428 (3)
C1—C6	1.392 (3)	C16—C17	1.367 (3)
C2—C3	1.375 (3)	C16—H16A	0.9300
C2—H2A	0.9300	C17—C18	1.399 (3)
C3—C4	1.408 (4)	C17—H17A	0.9300
C3—H3A	0.9300	C18—C19	1.377 (3)
C4—C5	1.373 (3)	C18—C21	1.501 (3)
C4—H4A	0.9300	C19—C20	1.417 (3)
C5—C6	1.381 (3)	C19—H19A	0.9300
C5—H5A	0.9300	C21—H21A	0.9600
C7—C8	1.468 (3)	C21—H21B	0.9600
C8—C9	1.406 (3)	C21—H21C	0.9600
			
O1—Zn1—N1	120.95 (7)	C10—C9—H9A	119.3
O1—Zn1—N3	95.00 (7)	C8—C9—H9A	119.3
N1—Zn1—N3	90.39 (7)	C11—C10—C9	119.6 (2)
O1—Zn1—Cl1	115.14 (5)	C11—C10—H10A	120.2
N1—Zn1—Cl1	111.84 (5)	C9—C10—H10A	120.2
N3—Zn1—Cl1	120.39 (6)	C10—C11—C12	120.7 (2)
C20—O1—Zn1	125.15 (14)	C10—C11—H11A	119.7
C7—N1—C1	106.20 (17)	C12—C11—H11A	119.7
C7—N1—Zn1	122.18 (15)	C11—C12—C13	120.6 (2)
C1—N1—Zn1	131.21 (13)	C11—C12—H12A	119.7
C7—N2—C6	108.47 (18)	C13—C12—H12A	119.7
C7—N2—H1N1	124 (2)	C12—C13—C8	119.72 (19)
C6—N2—H1N1	127 (2)	C12—C13—N3	121.3 (2)
C14—N3—C13	119.79 (18)	C8—C13—N3	118.95 (19)
C14—N3—Zn1	120.98 (14)	N3—C14—C15	126.9 (2)
C13—N3—Zn1	119.23 (14)	N3—C14—H14A	116.6
C2—C1—C6	121.4 (2)	C15—C14—H14A	116.6
C2—C1—N1	129.8 (2)	C16—C15—C20	119.0 (2)
C6—C1—N1	108.80 (18)	C16—C15—C14	116.5 (2)
C3—C2—C1	116.9 (2)	C20—C15—C14	124.3 (2)
C3—C2—H2A	121.6	C17—C16—C15	122.2 (2)
C1—C2—H2A	121.6	C17—C16—H16A	118.9
C2—C3—C4	121.4 (2)	C15—C16—H16A	118.9
C2—C3—H3A	119.3	C16—C17—C18	119.9 (2)
C4—C3—H3A	119.3	C16—C17—H17A	120.0
C5—C4—C3	121.8 (2)	C18—C17—H17A	120.0
C5—C4—H4A	119.1	C19—C18—C17	118.8 (2)
C3—C4—H4A	119.1	C19—C18—C21	120.3 (2)
C4—C5—C6	116.5 (2)	C17—C18—C21	120.9 (2)
C4—C5—H5A	121.7	C18—C19—C20	123.6 (2)
C6—C5—H5A	121.7	C18—C19—H19A	118.2
C5—C6—N2	132.6 (2)	C20—C19—H19A	118.2
C5—C6—C1	122.0 (2)	O1—C20—C19	118.2 (2)
N2—C6—C1	105.41 (19)	O1—C20—C15	125.38 (19)
N1—C7—N2	111.11 (19)	C19—C20—C15	116.4 (2)
N1—C7—C8	126.48 (19)	C18—C21—H21A	109.5
N2—C7—C8	122.33 (18)	C18—C21—H21B	109.5
C9—C8—C13	117.9 (2)	H21A—C21—H21B	109.5
C9—C8—C7	118.8 (2)	C18—C21—H21C	109.5
C13—C8—C7	123.31 (18)	H21A—C21—H21C	109.5
C10—C9—C8	121.5 (2)	H21B—C21—H21C	109.5
			
N1—Zn1—O1—C20	−107.74 (18)	N1—C7—C8—C9	152.4 (2)
N3—Zn1—O1—C20	−14.28 (18)	N2—C7—C8—C9	−24.0 (3)
Cl1—Zn1—O1—C20	112.73 (17)	N1—C7—C8—C13	−28.3 (3)
O1—Zn1—N1—C7	116.26 (16)	N2—C7—C8—C13	155.4 (2)
N3—Zn1—N1—C7	20.19 (17)	C13—C8—C9—C10	0.7 (3)
Cl1—Zn1—N1—C7	−103.02 (16)	C7—C8—C9—C10	−179.9 (2)
O1—Zn1—N1—C1	−55.4 (2)	C8—C9—C10—C11	0.7 (4)
N3—Zn1—N1—C1	−151.41 (18)	C9—C10—C11—C12	−1.2 (4)
Cl1—Zn1—N1—C1	85.37 (18)	C10—C11—C12—C13	0.3 (4)
O1—Zn1—N3—C14	13.98 (18)	C11—C12—C13—C8	1.1 (4)
N1—Zn1—N3—C14	135.10 (18)	C11—C12—C13—N3	−179.0 (2)
Cl1—Zn1—N3—C14	−109.09 (17)	C9—C8—C13—C12	−1.6 (3)
O1—Zn1—N3—C13	−166.00 (15)	C7—C8—C13—C12	179.0 (2)
N1—Zn1—N3—C13	−44.88 (16)	C9—C8—C13—N3	178.56 (19)
Cl1—Zn1—N3—C13	70.93 (16)	C7—C8—C13—N3	−0.8 (3)
C7—N1—C1—C2	179.7 (2)	C14—N3—C13—C12	40.8 (3)
Zn1—N1—C1—C2	−7.7 (3)	Zn1—N3—C13—C12	−139.24 (19)
C7—N1—C1—C6	−0.9 (2)	C14—N3—C13—C8	−139.4 (2)
Zn1—N1—C1—C6	171.74 (15)	Zn1—N3—C13—C8	40.6 (2)
C6—C1—C2—C3	0.7 (3)	C13—N3—C14—C15	174.0 (2)
N1—C1—C2—C3	−180.0 (2)	Zn1—N3—C14—C15	−6.0 (3)
C1—C2—C3—C4	−1.6 (3)	N3—C14—C15—C16	177.5 (2)
C2—C3—C4—C5	1.0 (4)	N3—C14—C15—C20	−7.2 (4)
C3—C4—C5—C6	0.7 (4)	C20—C15—C16—C17	−0.6 (4)
C4—C5—C6—N2	179.1 (2)	C14—C15—C16—C17	175.0 (2)
C4—C5—C6—C1	−1.6 (3)	C15—C16—C17—C18	−1.4 (4)
C7—N2—C6—C5	178.7 (2)	C16—C17—C18—C19	0.5 (4)
C7—N2—C6—C1	−0.7 (2)	C16—C17—C18—C21	179.3 (2)
C2—C1—C6—C5	1.0 (3)	C17—C18—C19—C20	2.6 (4)
N1—C1—C6—C5	−178.5 (2)	C21—C18—C19—C20	−176.2 (2)
C2—C1—C6—N2	−179.6 (2)	Zn1—O1—C20—C19	−174.38 (15)
N1—C1—C6—N2	1.0 (2)	Zn1—O1—C20—C15	6.2 (3)
C1—N1—C7—N2	0.4 (2)	C18—C19—C20—O1	176.0 (2)
Zn1—N1—C7—N2	−173.03 (14)	C18—C19—C20—C15	−4.5 (3)
C1—N1—C7—C8	−176.3 (2)	C16—C15—C20—O1	−177.2 (2)
Zn1—N1—C7—C8	10.3 (3)	C14—C15—C20—O1	7.6 (4)
C6—N2—C7—N1	0.2 (2)	C16—C15—C20—C19	3.4 (3)
C6—N2—C7—C8	177.05 (19)	C14—C15—C20—C19	−171.8 (2)

### Hydrogen-bond geometry (Å, °)

**Table:**

*D*—H···*A*	*D*—H	H···*A*	*D*···*A*	*D*—H···*A*
N2—H1N1···Cl1^i^	0.75 (3)	2.53 (3)	3.2352 (19)	157 (2)
C2—H2A···O1^ii^	0.93	2.59	3.425 (3)	149
C12—H12A···Cg1^iii^	0.93	2.96	3.762 (3)	145
C21—H21C···Cg2^iv^	0.96	2.92	3.741 (3)	144

Symmetry codes: (i) *x*+1, *y*, *z*; (ii) −*x*+1, −*y*, −*z*+1; (iii) *x*, −*y*−1/2, *z*−1/2; (iv) *x*, −*y*−1/2, *z*−3/2.

## References

[bb1] Allen, F. H., Kennard, O., Watson, D. G., Brammer, L., Orpen, A. G. & Taylor, R. (1987). *J. Chem. Soc. Perkin Trans. 2*, pp. S1–19.

[bb2] Bruker (2005). *APEX2*, *SAINT* and *SADABS* Bruker AXS Inc., Madison, Wisconsin, USA.

[bb3] Chassaing, C., Berger, M., Heckeroth, A., IIg, T., Jaeger, M., Kern, C., Schmid, K. & Uphoff, M. (2008). *J. Med. Chem* **51**, 1111–1114. 10.1021/jm701456r 18271517

[bb4] Cosier, J. & Glazer, A. M. (1986). *J. Appl. Cryst.* **19**, 105–107.

[bb5] Eltayeb, N. E., Teoh, S. G., Chantrapromma, S. & Fun, H.-K. (2007). *Acta Cryst.* E**63**, o4141–o4142.

[bb6] Eltayeb, N. E., Teoh, S. G., Chantrapromma, S. & Fun, H.-K. (2011). *Acta Cryst.* E**67**, m1062–m1063. 10.1107/S1600536811026572 PMC3212144 22090846

[bb7] Eltayeb, N. E., Teoh, S. G., Quah, C. K., Fun, H.-K. & Adnan, R. (2009). *Acta Cryst.* E**65**, o1613–o1614. 10.1107/S1600536809022478 PMC2969490 21582885

[bb8] Eltayeb, N. E., Teoh, S. G., Yeap, C. S. & Fun, H.-K. (2011). *Acta Cryst.* E**67**, o1721–o1722. 10.1107/S160053681102304X PMC3152048 21837112

[bb9] Kucukbay, H., Durmaz, R., Orhan, E. & Gunal, S. (2003). *Farmaco*, **58**, 431–437. 10.1016/S0014-827X(03)00068-5 12767382

[bb10] Maldonado-Rogado, M. A., Viñuelas-Zahínos, E., Luna-Giles, F. & Bernalte-García, A. (2007). *Polyhedron*, **26**, 3112–3120.

[bb11] Podunavac-Kuzmanovic, S. & Cvetkovic, D. (2010). *Rev. Roum. Chim* **55**, 363–367.

[bb12] Podunavac-Kuzmanovic, S. O., Leovac, L. M., Perisic-Janjic, N. U., Rogan, J. & Balaz, J. (1999). *J. Serb. Chem. Soc* **64**, 381–388.

[bb13] Podunavac-Kuzmanovic, S. O. & Markov, S. L. (2006). *Centr. Eur. J. Occupat. Environ. Med* **12**, 61–66.

[bb14] Sheldrick, G. M. (2008). *Acta Cryst.* A**64**, 112–122. 10.1107/S0108767307043930 18156677

[bb15] Spek, A. L. (2009). *Acta Cryst.* D**65**, 148–155. 10.1107/S090744490804362X PMC2631630 19171970

[bb16] Tong, Y.-P. & Ye, B.-H. (2004). *Acta Cryst.* E**60**, m1927–m1929.

[bb17] Xue, F., Luo, X., Ye, C., Ye, W. & Wang, Y. (2011). *Bioorg. Med. Chem* **19**, 2641–2649. 10.1016/j.bmc.2011.03.007 21441033

